# Preliminary Evaluation of Dominant Hand Mimicry Versus Traditional Grip Training for Non-dominant Hand Chopstick Use: A Single-Case Design

**DOI:** 10.7759/cureus.103375

**Published:** 2026-02-10

**Authors:** Yuwen Fu, Yuki Choji

**Affiliations:** 1 Department of Motor Function Science, Graduate School of Rehabilitation, Niigata University of Rehabilitation, Murakami, JPN; 2 Department of Physical Medicine and Rehabilitation, Faculty of Allied Health Science, Niigata University of Rehabilitation, Murakami, JPN

**Keywords:** chopstick use, dominant hand-mimicking training, fine motor skills, non-dominant hand, rehabilitation, single-case design, traditional training

## Abstract

Background: Many patients with stroke lose functional use of their hands, highlighting the importance of non-dominant hand training as a compensatory strategy in rehabilitation. Chopstick manipulation training is commonly practiced in East Asia; however, it remains unclear whether a traditional grip or a dominant hand-like grip is more effective for training the non-dominant hand.

Methods: Ten right-handed students (five Japanese, five Chinese; eight males, two females; aged 20-26 years) participated in this study. Participants trained with their non-dominant hand for 15 minutes per day over 10 days using an alternating treatments design (ATD), while viewing instructional videos and concurrently practicing chopstick tip alignment exercises with either the traditional grip (Intervention A) or a dominant hand-like grip (Intervention B). Outcomes included the number of bean transfers within two minutes, chopstick tip traction strength, grip style (Yokubo classification), and subjective sense of accomplishment.

Results: All 10 participants completed the training with no dropouts. Analysis indicated weak to strong effects of Intervention B on bean transfers (three of five Chinese and all five Japanese) and chopstick tip traction force (three of five Chinese and four of five Japanese). After Intervention A, all participants adopted the “Four Finger” grip, with subjective achievement generally moderate to high. After Intervention B, both groups shifted toward “Three Finger” or “Other” grips, with subjective achievement remaining medium to high.

Conclusions: Grip style plays a critical role in non-dominant hand training. For some tasks, adopting a grip similar to the dominant hand may enhance functional performance and offer practical benefits for stroke rehabilitation.

## Introduction

The number of stroke patients worldwide is increasing, and as of 2019, the total number has been estimated at approximately 101 million [1,2]. Among them, approximately 33% to 66% experience difficulty in recovering hand function and ultimately develop disuse of the affected hand [3-5]. This highlights the need for handedness-switch training, particularly for culturally important tasks such as using chopsticks and writing. Similar to functional recovery training, establishing evidence for compensatory strategies through handedness change is essential in stroke rehabilitation.

Chopstick training is a typical example of handedness-switch training. The traditional chopstick grip is generally recommended for its ease of use and etiquette [6-9]. However, comparative studies examining differences among grip styles are lacking. Most published research has focused only on comparisons with the “scissors-pinching” grip [6-8,10,11]. In scissors-pinching, two chopsticks are crossed between the index and middle fingers, stabilized by the tips of the middle and ring fingers, and moved by applying pressure with the thumb and index finger. This is classified as a type 1 lever. By contrast, the traditional grip - holding the upper chopstick between the thumb, index, and middle fingers while securing the lower chopstick between the base of the thumb and the ring finger [12,13] - is classified as a type 3 lever [10]. This traditional grip, also referred to as pincers-pinching, has been shown to be more accurate than scissors-pinching [6-8]. Nevertheless, as dozens of alternative grips have been reported [14], further comparative studies of the traditional grip with other styles are warranted.

In clinical practice, support for chopstick grip training is tailored to each individual’s preferences, age, dexterity, and cognitive status [15], although the traditional grip is often recommended. Yet, acquiring the traditional grip with the non-dominant hand is challenging due to its complexity. A review by Choji et al. [16] described various approaches for non-dominant hand chopstick training, including operational pattern classification, tip alignment training, finger ball rolling, and the use of physical guidance or assistive devices. However, none of these specifically targeted a particular grip style. In contrast, Yamada et al. [17] investigated whether chopstick grips differed between dominant and non-dominant hands. They asked 38 healthy participants to transfer ping-pong balls using both hands and found inconsistencies in grip patterns, with especially reduced interdigital separation in the non-dominant hand. These findings suggest that assistance for non-dominant hand use may need to account for differences in interdigital separation, but it remains unclear whether training can facilitate a grip similar to the dominant hand. Moreover, as these findings are based on a limited sample in Japan, further research is needed that considers cultural differences across countries.

Therefore, this study aimed to compare the effectiveness of a traditional grip and a dominant hand-like grip in non-dominant hand chopstick training, examining their impact on functional performance, grip style, and subjective accomplishment.

## Materials and methods

Participants

Ten undergraduate and graduate students from our university participated in the study (five Japanese, five Chinese; eight males, two females; age range 20-26 years, mean 21.9 ± 1.66 years). All participants were right-handed; none were left-handed. According to Chambless and Hollon [18], the level of evidence for empirically supported treatments for single-case design (SCD) is classified as “probably efficacious” when three or more participants are involved, and as “well-established” when nine or more are included. Based on this framework, the sample size for the present study was determined. Inclusion criteria were participants who did not use the traditional chopstick grip with their dominant hand. In this study, the traditional grip was defined according to the criteria of Mukai and Isshiki [12,13]: holding the upper chopstick with the first, second, and third fingers, and securing the lower chopstick between the first and fourth fingers. The contact point of the lower chopstick with the fourth finger was not limited to the fingertip (side of the nail). Exclusion criteria were (1) inability to hold chopsticks due to injury or disability in the dominant hand; (2) previous experience with handedness-switch training; and (3) inability to understand the study purpose. This study was approved by the Ethics Committee of Niigata Rehabilitation University (Niigata, Japan) on October 22, 2024 (Approval No. 262).

Design

This study employed an alternating treatments design (ATD), a type of SCD.

Procedure

After informed consent was obtained, participants’ handedness was first assessed using the Edinburgh Handedness Test [19]. Two interventions were then administered: Intervention A (traditional chopstick grip) and Intervention B (grip similar to the dominant hand). These were applied over a 10-day period following the ATD framework. A block randomization method was used to ensure an equal number of sessions with Intervention A and Intervention B (five days each). Previous studies have reported highly variable training durations for chopstick use in healthy individuals, ranging from three minutes to four weeks [16]. Therefore, in consideration of the number of data points required for ATD analysis [20], the duration of each intervention was set to five days.

Intervention method

Intervention A trained participants to hold chopsticks in the traditional manner with their non-dominant hand, whereas Intervention B trained them to hold chopsticks in a manner similar to their dominant hand. Both training methods consisted of 15 minutes of chopstick tip alignment training while watching an instructional video on an iPad (Apple Inc., Cupertino, CA, USA). Chopstick tip alignment training involved confirming the correct grip and then repeatedly opening and closing the chopsticks while ensuring that the tips did not cross [21]. Instructional videos were recorded in advance as follows: in Intervention A, the researcher held chopsticks in the traditional manner with the dominant hand and performed opening and closing movements for 30 seconds (at a rate of two times per second). In Intervention B, each participant demonstrated their usual manner of holding chopsticks with their dominant hand. The forearm and wrist were filmed in a neutral position. The videos were presented on an Apple iPad Air 2 (9.7-inch Retina display, A8X chip, 8MP rear camera).

Chopsticks

**Figure 1:**
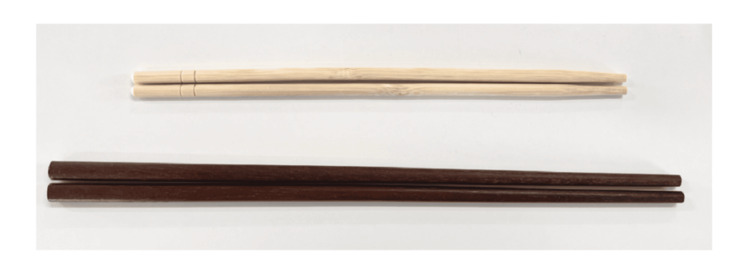
Two types of chopsticks. Top: Japanese chopsticks; Bottom: Chinese chopsticks.

In Japan, bamboo or wooden chopsticks are commonly used, and the recommended length is approximately 1.2 times the length of the palm [13]. In China, wooden chopsticks are typically preferred, with an average length of 25 cm, which is longer than those used in Japan [13]. Differences also exist in weight and shape. Considering these cultural backgrounds and the experimental environment, we prepared two types of chopsticks that are readily available for everyday use in Japan and China (Figure 1). The Japanese chopsticks were bamboo (200 mm in length, 6 g in weight), while the Chinese chopsticks were wooden (250 mm in length, 18 g in weight).

Outcome

Three main outcomes were measured: (1) the number of adzuki beans (a type of small red bean commonly used in East Asia) transferred between the plates, (2) the traction force at the chopstick tips, and (3) the chopstick grip style and subjective achievement.

Adzuki Bean Transfer Task

**Figure 2:**
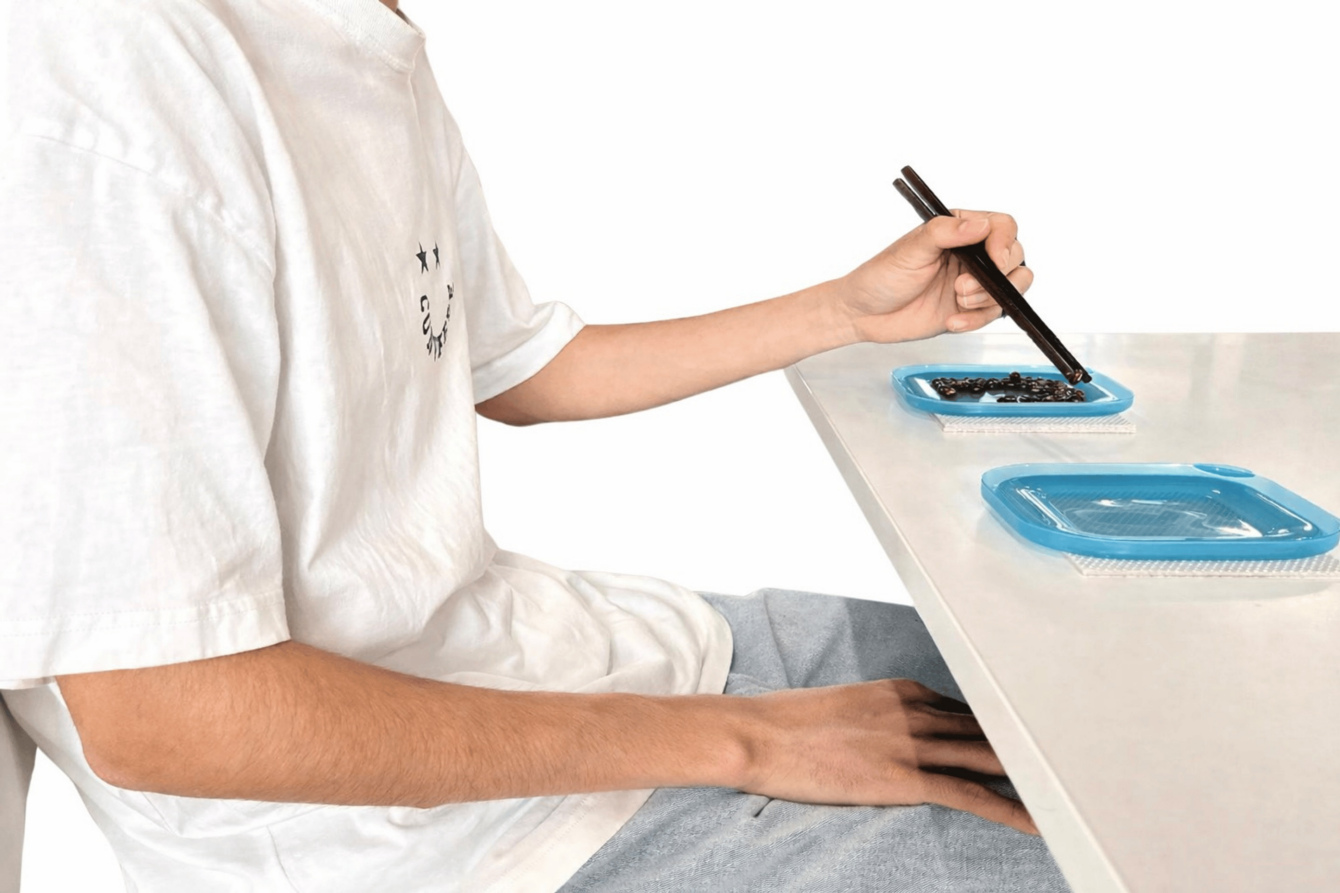
Number of adzuki beans transferred between plates.

This task was based on the experimental setup described by Takeda and Miyamoto [22]. Two plates (125 mm × 125 mm × 10 mm) were prepared, and 70 adzuki beans were placed on the left plate. The distance between the plates was 100 mm, and the distance from the participant to the plates was 450 mm (Figure 2). Participants began with their dominant hand resting on their lap and their non-dominant hand holding chopsticks. At the examiner’s signal, they transferred the beans from the left to the right plate as quickly as possible within two minutes. If beans were dropped outside the plates, they were instructed to leave them. After the task, the number of successfully transferred and dropped beans was recorded.

Traction Force at the Chopstick Tips

The traction force was measured using a push-pull scale (Imada Standard Mechanical Force Gauge, PS-20N; Imada Co., Ltd., Toyohashi, Japan), following the method of Hsu and Wu [6,10]. An eraser (40 mm × 25 mm × 15 mm) was fixed to the clasp of the device, and participants pinched it with the chopstick tips while pulling toward themselves for three seconds to measure maximum traction force. Participants began with both forearms pronated and resting on the table. Measurements were taken three times, and the average was used.

Grip Style and Subjective Achievement

At the beginning and end of Interventions A and B, chopstick grip style was evaluated according to the classification by Yokubo et al. [23], and contact points between the chopsticks and fingers were recorded. Yokubo et al. classified grip styles into three categories based on the contact points between the upper and lower chopsticks and the fingers: “Four Finger,” “Three Finger,” and “Palm.” Grips that did not fit into these categories were classified as “Other.” Finally, participants rated their subjective sense of achievement regarding whether they had successfully mastered holding chopsticks on a 5-point Likert scale, ranging from “Strongly disagree” to “Strongly agree.”

Statistics

For all participants, the number of adzuki beans transferred and the chopstick tip traction force under Interventions A and B were graphed and analyzed using visual inspection and trend analysis. For visual inspection, the level was calculated as the median, and the trend was estimated using the least squares method. Effect sizes were calculated using the weighted percentage of non-overlapping data (PND-W), a method specifically developed for ATD [24,25]. According to the evaluation criteria of Scruggs and Mastropieri [26], PND scores were interpreted as follows: ≥90% indicates a strong effect, 70-89% a moderate effect, 50-69% a weak effect, and <50% no effect. Descriptive analyses were also conducted for the initial and final results of Interventions A and B. All analyses were performed using an SCD web application [27].

## Results

All 10 participants completed the study without missing data.

Number of adzuki beans transferred between plates

**Figure 3:**
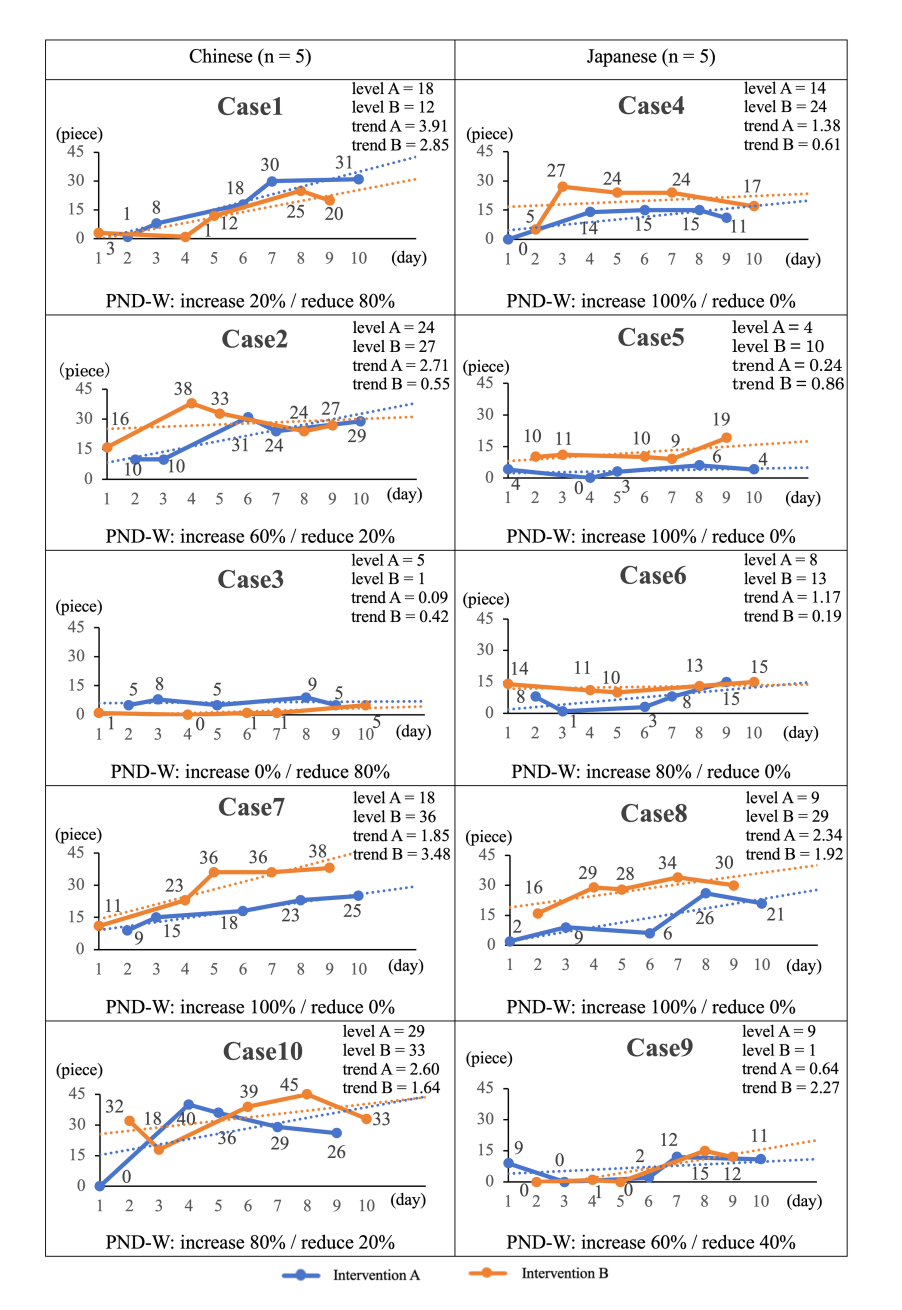
Ten-day changes in adzuki bean transfers (two minutes) by Chinese and Japanese participants under Interventions A and B. PND-W: weighted percentage of non-overlapping data; Level: calculated as the median; Trend: calculated using the least-squares method. According to the evaluation criteria of Scruggs and Mastropieri [26], PND scores were interpreted as follows: ≥90% indicates a strong effect, 70-89% a moderate effect, 50-69% a weak effect, and <50% no effect.

For Chinese participants, the level of beans transferred was higher in Intervention B for three of five participants, and the trend was higher for two of five participants. The results were as follows: Intervention A, level range 5-29 beans, trend range 0.09-3.91; Intervention B, level range 1-36 beans, trend range 0.42-3.48. According to PND-W, three of five participants showed effects of Intervention B ranging from weak to strong. For Japanese participants, the level of beans transferred was higher in Intervention B for four of five participants, and the trend was higher for two of five participants. The results were as follows: Intervention A, level range 4-14 beans, trend range 0.24-2.34; Intervention B, level range 1-29 beans, trend range 0.19-2.27. Based on PND-W, all five participants showed effects of Intervention B ranging from weak to strong (Figure 3).

Chopstick tip traction force

**Figure 4:**
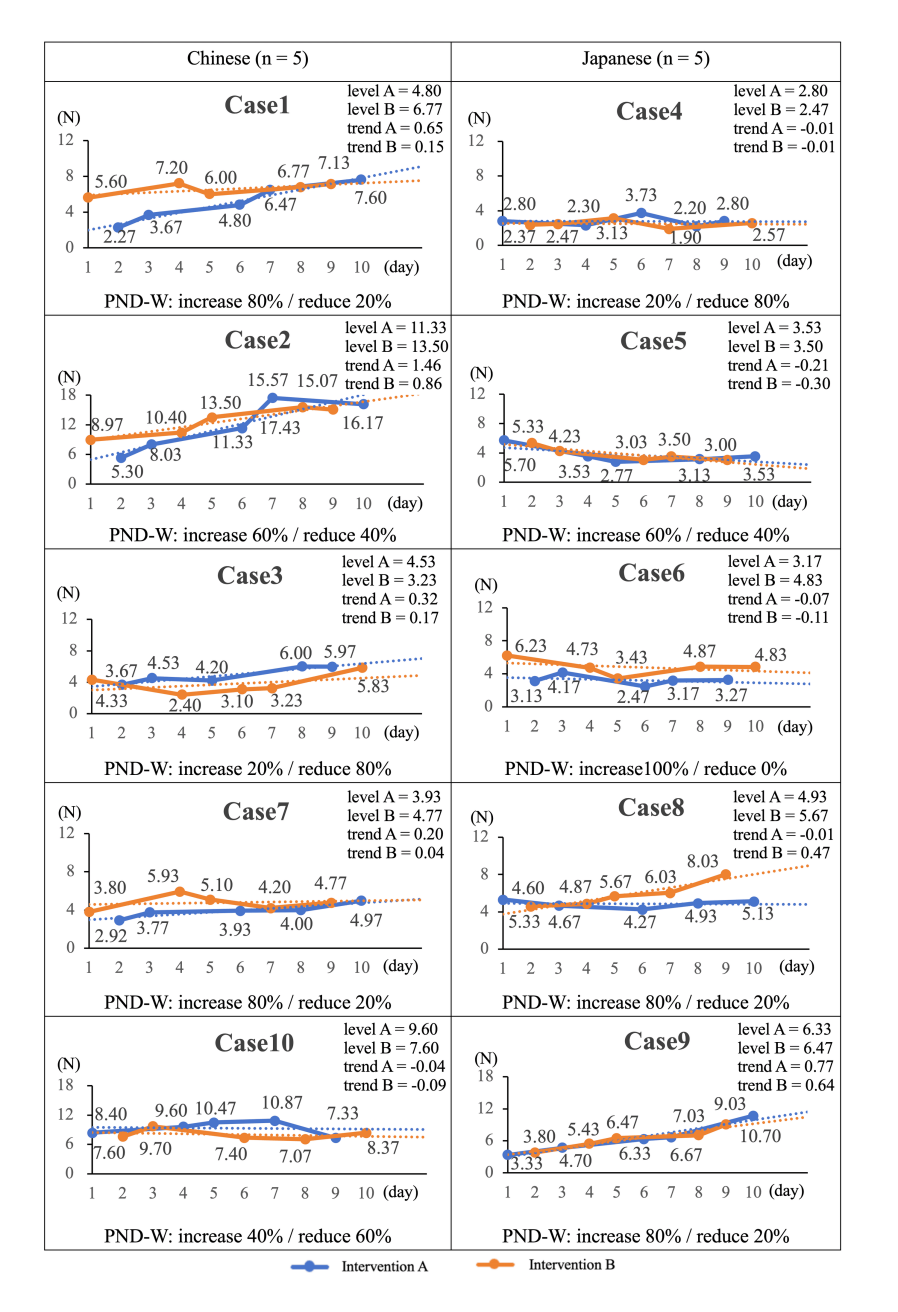
Ten-day changes in chopstick tip traction force by Chinese and Japanese participants under Interventions A and B. PND-W: weighted percentage of non-overlapping data; Level: calculated as the median; Trend: calculated using the least-squares method. According to the evaluation criteria of Scruggs and Mastropieri [26], PND scores were interpreted as follows: ≥90% indicates a strong effect, 70-89% a moderate effect, 50-69% a weak effect, and <50% no effect.

For Chinese participants, the traction force at the chopstick tips was higher with Intervention B in three of five participants, whereas the trend values were higher with Intervention A in all five participants. The results were as follows: Intervention A, level range 3.93-11.33 N and trend range -0.04 to 1.46; Intervention B, level range 3.23-13.50 N and trend range -0.09 to 0.86. Based on PND-W, three of five participants showed effects of Intervention B ranging from weak to moderate. For Japanese participants, Intervention B showed higher levels in three of five participants and higher trends in one of five. The results were as follows: Intervention A, level range 2.80-6.33 N and trend range -0.21 to 0.77; Intervention B, level range 2.47-6.47 N and trend range -0.30 to 0.64. According to PND-W, four of five participants showed effects of Intervention B ranging from weak to strong (Figure 4).

Comparison of chopstick grip methods and subjective achievement before and after interventions

After Intervention A, all Chinese and Japanese participants demonstrated a “Four Finger” grip according to Yokubo et al.’s classification. For subjective achievement, four of five Chinese participants rated 5 points, and one rated 4 points. Among Japanese participants, two of five rated 5 points, two rated 4 points, and one rated 3 points. After Intervention B, three Chinese participants demonstrated a “Three Finger” grip, and two were classified as “Other.” Among Japanese participants, three demonstrated a “Three Finger” grip, and two were classified as “Other.” For subjective achievement, three of five Chinese participants rated 5 points, one rated 4 points, and one rated 3 points. Among Japanese participants, two of five rated 5 points, and three rated 4 points. For a comprehensive overview of the study findings, please refer to Appendices A-B.

## Discussion

Azuki bean transfer and chopstick grip methods

For azuki bean transfer, Intervention B showed effects ranging from weak to strong in three of five Chinese participants and in all five Japanese participants. These findings suggest that, although individual differences were observed among Chinese participants, Intervention B was consistently more effective than Intervention A among Japanese participants. Chopstick grip methods and subjective achievement scores before and after the interventions (Appendices A-B) showed that all Japanese participants adopted the “Four Finger” grip, indicating that the traditional grip had been acquired to some extent. Previous studies [6,11,13] have also reported that the stability of the proximal chopstick is important for mastering the traditional grip. The fact that all Japanese participants achieved this stability in the present study may have contributed to the formation of the grip during Intervention A. However, the bean transfer task required delicate force control and rapid manipulation. Intervention B, which involves relatively less finger separation and is thought to facilitate stable manipulation, may have enabled more efficient performance under these conditions [6,9]. There was considerable variability in performance: 5-29 transfers in Intervention A and 1-36 transfers in Intervention B among Chinese participants, and 4-14 transfers in Intervention A and 1-29 transfers in Intervention B among Japanese participants. Choji et al. [28] reported an average of 38.2 ± 11.3 successful transfers in a two-minute soybean-transfer task among 10 healthy adults, substantially higher than the present results. Although this study mainly compared Interventions A and B based on effect size, several factors, such as measurement error, participant characteristics, and task familiarity, must also be considered. Differences in chopstick and bean materials may also influence performance. Yang et al. [29] reported that grip accuracy differs between plastic and metal chopsticks, although the difference is small for wooden chopsticks. Similarly, Mukai and Hashimoto [12] compared chopsticks from China, Japan, and Korea and concluded that, except for Korean chopsticks, differences in length and weight had limited effects on task completion. From this perspective, the difference between the wooden and bamboo chopsticks used in this study may have had only a minor influence. Future studies under more controlled conditions are warranted.

Chopstick tip traction force

For chopstick tip traction force, Intervention B showed effects ranging from weak to moderate in three out of five Chinese participants, and from weak to strong in four out of five Japanese participants. By grip style, the average traction force was 6.81 N for the “Three Finger” grip, 5.50 N for the “Four Finger” grip, and 4.49 N for “Other.” Previous studies [6-9] have reported that the “Three Finger” grip more effectively utilizes palmar grip force and generates stronger force than the “Four Finger” grip. The present results support these findings. For Chinese participants, the traction force averaged 6.84 N in Intervention A and 7.17 N in Intervention B, while for Japanese participants, the values were 4.15 N and 4.59 N, respectively. Thus, Chinese participants showed higher traction force in both conditions. Chopstick shape may be one factor. Yokubo et al. [23] reported that the efficiency of force transmission to the chopstick tips varies depending on handle shape, with square chopsticks exerting greater force than octagonal or triangular ones. Accordingly, the square-shaped chopsticks used by Chinese participants may have provided a flatter grip, greater stability, and better transmission efficiency than the round-shaped chopsticks used by Japanese participants, facilitating greater force application at the tips.

Limitations and future directions

A major strength of this study lies in its cross-cultural perspective, as it included participants from different cultural backgrounds. This diversity enriches the interpretation of the results and allows for meaningful cross-cultural comparisons of chopstick gripping methods. Such findings may contribute to developing culturally sensitive rehabilitation strategies for hand function recovery and to educational programs aimed at enhancing fine motor skills across populations. In addition, the study provides preliminary evidence supporting the feasibility of handedness-switch training using chopsticks, a culturally and functionally relevant activity in many Asian societies. This approach has potential implications for stroke rehabilitation, where patients often experience disuse of the affected hand. Training the non-dominant hand to acquire skilled, culturally meaningful movements could enhance motivation and promote engagement in everyday activities.

Several limitations should also be noted. First, the sample size was small (n = 10) and limited to healthy university students, restricting generalizability to clinical populations. Second, although the intervention period was 10 days, details of the instructions, feedback, and physical setting were not fully described, which may limit reproducibility. Third, chopstick grip style was classified qualitatively using the method of Yokubo et al.; quantitative kinematic analyses are warranted. Fourth, the chopstick material and cultural background were not controlled, so their potential influence cannot be excluded.

Future research should extend the training period, include stroke survivors and other clinical populations, and examine how handedness-switch training affects daily living and neural plasticity. Furthermore, standardized training protocols, objective motor performance measures, and detailed cultural analyses will be essential for advancing the evidence base. These efforts will deepen understanding of handedness-switch learning mechanisms and inform practical approaches for culturally grounded rehabilitation.

## Conclusions

This study compared two types of non-dominant hand training for acquiring chopstick manipulation skills: holding chopsticks in the traditional way (Intervention A) and holding chopsticks in a manner similar to the dominant hand (Intervention B). The results suggest that Intervention B was effective for some participants in terms of movement accuracy and chopstick tip traction force. In particular, for tasks requiring rapid manipulation, a grip style similar to that of the dominant hand may be more practical. These findings highlight the importance of selecting an appropriate grip style and providing individualized support in non-dominant hand training, with potential applications in clinical practice and educational settings.

## Figures and Tables

**Figure 5:**
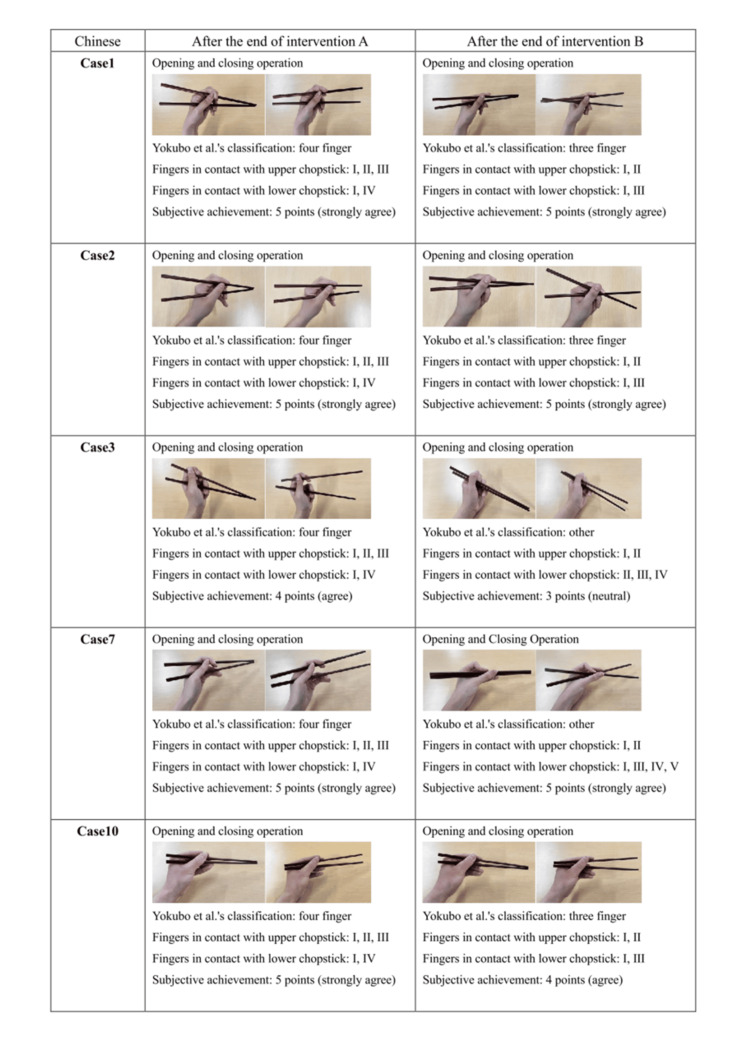
Chopstick grip methods and subjective achievement scores before and after the interventions among Chinese participants. The classification method for holding chopsticks is based on the method of Yokubo et al. [23].

**Figure 6:**
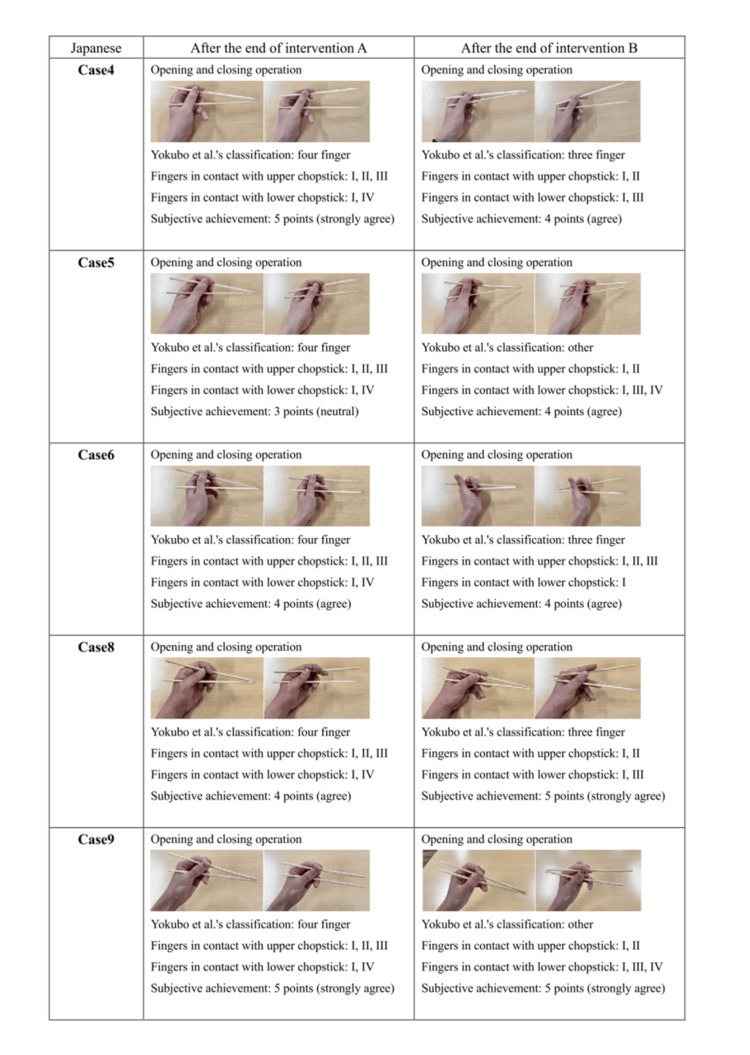
Chopstick grip methods and subjective achievement scores before and after the interventions among Japanese participants. The classification method for holding chopsticks is based on the method of Yokubo et al. [23].

## Appendices

Appendix A 

Appendix B 
